# Acupuncture Improves Sleep Conditions of Minipigs Representing Diurnal Animals through an Anatomically Similar Point to the Acupoint (GV20) Effective for Humans

**DOI:** 10.1155/2012/472982

**Published:** 2012-02-16

**Authors:** Ka-ichiro Takeishi, Masahisa Horiuchi, Hiroaki Kawaguchi, Yoshiki Deguchi, Hiroyuki Izumi, Emi Arimura, Satoshi Kuchiiwa, Akihide Tanimoto, Toru Takeuchi

**Affiliations:** ^1^Department of Environmental Medicine, Graduate School of Medical and Dental Sciences, Kagoshima University, 8-35-1 Sakuragaoka, Kagoshima 890-8544, Japan; ^2^Department of Veterinary Pathology, Faculty of Agriculture, Kagoshima University, 1-21-24 Korimoto, Kagoshima 890-0065, Japan; ^3^Drug Safety Research Laboratories, Shin Nippon Biomedical Laboratories, Ltd., 2438 Miyanoura, Kagoshima 891-1394, Japan; ^4^Department of Life and Environmental Science, Kagoshima Prefectural College, 1-52-1 Shimo-Ishiki, Kagoshima 890-0005, Japan; ^5^Department of Neuroanatomy, Graduate School of Medical and Dental Sciences, Kagoshima University, 8-35-1 Sakuragaoka, Kagoshima 890-8544, Japan; ^6^Department of Molecular and Cellular Pathology, Graduate School of Medical and Dental Sciences, Kagoshima University, 8-35-1 Sakuragaoka, Kagoshima 890-8544, Japan

## Abstract

Acupuncture, an alternative medicine, has been widely applied for people with sleep disturbances; therefore, the effects should be evaluated objectively. Micro-minipigs (MMPigs), the smallest miniature pigs for animal experiments, were used. Acupuncture was performed at two different points: Dafengmen is located on the head and is an anatomically similar point to human-Baihui (GV20), an effective acupoint for sleep disturbances in humans; pig-Baihui is on the back. The procedure was performed as follows: shallow, within 5 mm depth for several seconds; deep, 10–20 mm depth for 20 min. The sleep conditions were evaluated by actigraph, and the amount of catecholamine in pooled urine after acupuncture treatment. MMPigs with deep acupuncture at Dafengmen showed significantly efficient values on actigraph and catecholamine analysis as compared with untreated MMPigs. The effective acupoint for sleep conditions in the porcine model is at an anatomically similar point to humans, rather than the point determined by traditional Chinese medicine.

## 1. Introduction

People with sleep disturbances have increased in developed countries [1, 2]. Not only aged people but also working people often show sleep disturbances, causing severe problems in industry [3, 4]. Additionally, from the viewpoint of medical economics, the costs of treating sleep disturbances have stressed the government budget, so efficient treatment for sleep disturbance is required [3, 4].

Pharmacological drugs involved in serotonin and melatonin metabolism have been applied for people with sleep disturbances [5, 6]; however, the number of people with sleep disturbances has not tended to decrease. At present, not only pharmacological drugs but also alternative medicines are administered [7]. Acupuncture, an alternative medicine, is believed to relieve sleep disturbances [8–10]. In clinical studies, most results are evaluated by interviewing acupuncture subjects [8–10], and there is little objective evidence to reveal the effects of acupuncture. Here, an actigraph, an objective indicator evaluating the quantity and quality of sleep, was applied to an animal model. Placebo-controlled trial models do not work well for most nonpharmacological treatments, including acupuncture, because they tend to lack credible placebo controls [11–13]. Subjects receiving acupuncture might have information about its effects, leading to subjective effects of the procedure in humans. On the other hand, animals do not have information about the procedure; therefore, animal models are beneficial for research into acupuncture for insomnia. Some studies on rodents have been reported; however, their extrapolation to humans is restricted due to the difference in sleep patterns between rodents and humans [14].

As large mammals, monkeys have been used for sleep analysis, showing a similar sleep pattern to humans and higher diurnal activity than rodents [15–17]; however, it may be labor-intensive to evaluate the effects of acupuncture on monkeys because of the handling difficulty. On the other hand, pigs as domestic animals can be treated easily by acupuncture. Moreover, there is some information on the relationships between acupoints and complaints [18–20]; however, adult pigs have not been fully examined under sleep conditions [21, 22]. Micro-minipigs (MMPigs), a type of miniature pig, are a new porcine model showing atherosclerosis with a high-fat diet [23, 24]. In order to evaluate the effects of acupuncture on sleep conditions, MMPigs should be examined under sleep conditions.

Here, we examined whether MMPigs show a similar sleep pattern to humans. Moreover, the effects of acupuncture on sleep were evaluated objectively by an actigraph and urinary catecholamine [25–29]. We have tried to establish an animal model to evaluate objectively the effects of alternative medicines on sleep conditions.

## 2. Methods

### 2.1. Animals

MMPigs were obtained from the breeder (Fuji Micra Inc., Shizuoka, Japan) and kept in a humidity- and temperature-controlled (50 ± 10%, 22 ± 2°C) facility with a 12 h light/dark cycle (0700 h, lights-on) of Shin Nippon Biomedical Laboratories (Kagoshima, Japan) certified by the Association for the Assessment and Accreditation of Laboratory Animal Care. Light intensity during the day was around 400 lx. Food (ET; Nosan Co., Yokohama, Japan) was supplied to the pigs at around 1600 h. The pigs had free access to water. This study was approved by the Ethics Committee for Animal Experimentation at the Kagoshima University.

### 2.2. Acupuncture

**Figure 1:**
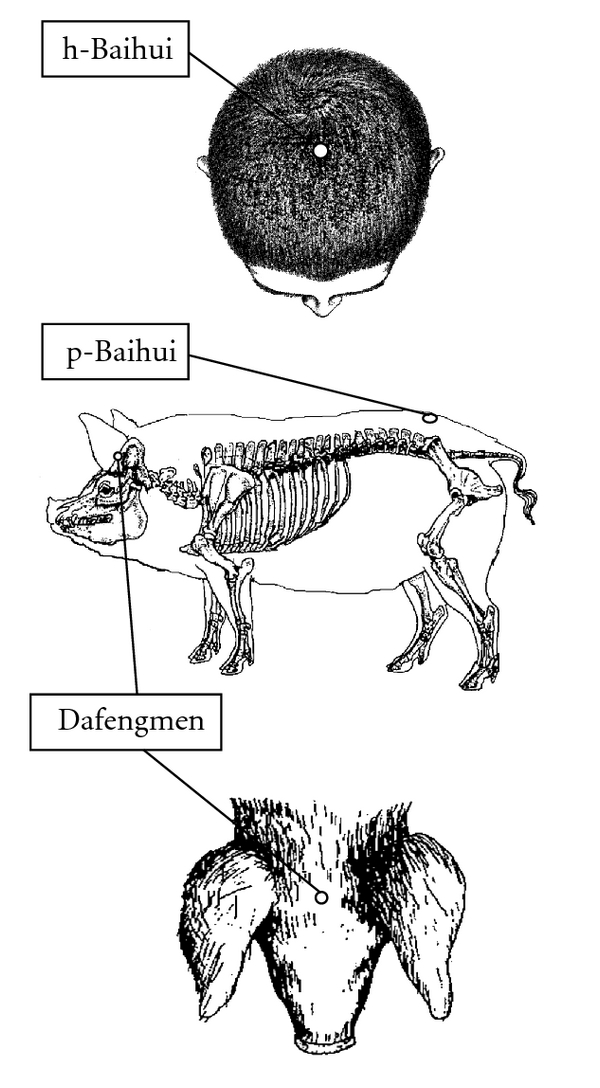
Acupoints for pigs used in the present study are shown in comparison with human acupoints. Human-Baihui (h-Baihui, GV20), the midpoint of a line connecting both ears, is an anatomically similar point to Dafengmen in pigs. Pig-Baihui (p-Baihui) is on top of the body, which designates the point of Baihui according to traditional Chinese medicine.

**Figure 2:**
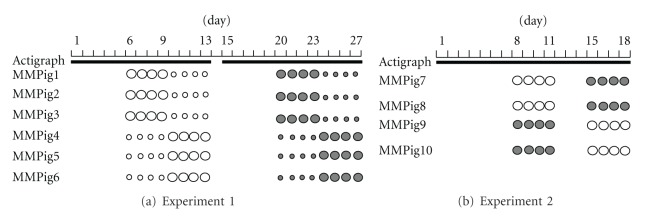
The schedule of acupuncture for experiments 1 and 2 is shown. Solid line means the time when the pig was equipped with the actigraph. Open and closed circles mean the acupuncture procedure performed at p-Baihui and Dafengmen (see Section 2), respectively. Small and large circles mean shallow and deep procedures (see Section 2), respectively.

Subjects assigned to acupuncture were punctured at Dafengmen and pig-Baihui (p-Baihui) according to traditional Chinese veterinary medicine (Figure 1) [18–20]. Dafengmen is located at the midpoint of a line connecting both ears viewed from overhead, which is anatomically similar to the acupoint human-Baihui (h-Baihui, Governor Vessel 20: GV20). Baihui is designated as the vertex on the midline based on traditional Chinese medicine [18–20]; therefore, p-Baihui is located at the top of the back near the hip, as shown in Figure 1 [18]. The syringes (diameter × length: 0.2 × 50 mm, J type) were obtained from Seirin Co. (Shizuoka, Japan). The acupuncture procedures were as follows: shallow method, the insertion depth was within 5 mm for several seconds; deep method, 10 mm at Dafengmen and 20 mm at p-Baihui for 20 min. The acupuncture procedures were performed at around 1730–1800 h. Two experiments were performed, as shown in Figure 2. For the first experiment, 6 male pigs (MMPigs 1–6), one year old, ranging from 8.0 to 10.0 kg body weight, were used. During days 6–13, MMPigs were treated at p-Baihui with the deep or shallow procedure. During days 20–27, MMPigs were treated at Dafengmen with the deep or shallow procedure. For the second experiment to evaluate urinary catecholamine, 4 male pigs (MMPigs 7–10), aged 6 months, ranging from 6.0 to 8.0 kg body weight, were used. During days 8–11 and days 15–18, MMPigs were treated at p-Baihui or Dafengmen with the deep procedure.

### 2.3. Actigraph

An actigraph (Octagonal Basic Motionlogger; Ambulatory Monitoring, Inc., Ardsley, NY, USA) was worn 24 h/d on the body of the pigs for 2-3 consecutive weeks (Figure 2) [25, 26]. We used Zero-Crossing Mode for quantification of movement with ACTION-W 2.0 software. As parameters for sleep conditions, total sleep time (TST), sleep efficiency (SE), and actual activity counts were according to an algorithm based on the human algorithm. We analyzed three or four consecutive days' data for the respective treatment [30, 31].

### 2.4. Urinary Analysis

In the metabolic cages, MMPigs were housed on metal slats, so that urine could be collected in containers. Urine was collected from 1900 to 0900 h on days 2–4, days 8–11, and days 15–18 in experiment 2 (Figure 2). The urine was centrifuged, and then 1 mL of 0.1 mol/L EDTA was added to 10 mL urine [27–29]. Thereafter, the supernatant was frozen and stored at −20°C. Norepinephrine and epinephrine were determined with solid-phase extraction and HPLC with electrochemical detection. The values are expressed as the means of the amount excreted (concentration (mg/mL) × urine volume for 14 h (mL)) of the urine collected.

### 2.5. Data Analysis

Values are shown as the respective data. Statistical analysis was performed using the paired Student's *t*-test. *P* < 0.05 was considered significant.

## 3. Results

### 3.1. Actigraph

**Figure 3:**
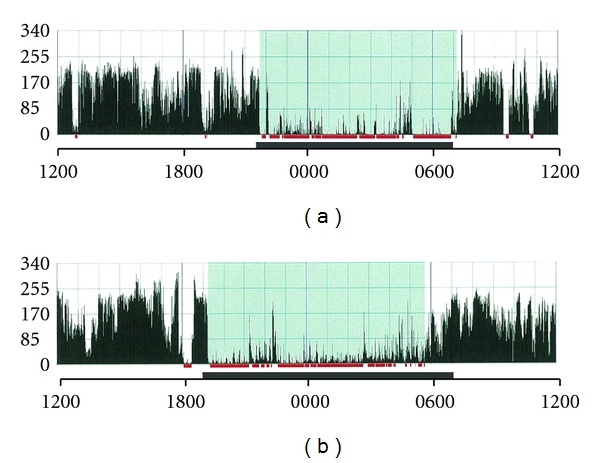
Representative data of actigraph for one day in humans (a) and MMPigs (b). Longitudinal axis means activity counts per minute. Solid horizontal box means the duration of lights off. According to the algorithm for human data analysis, the blue region means lying on the side, and the red mark means sleeping.

**Figure 4:**
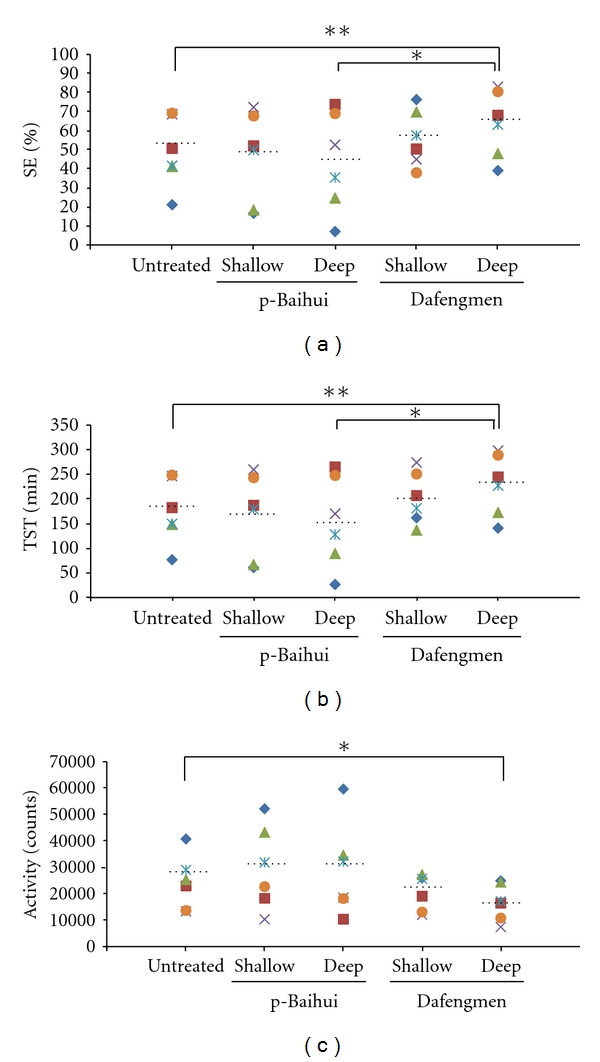
Effects of acupuncture on sleep conditions including sleep efficiency (SE, (a)), total sleep time (TST, (b)), and activity (c) during 2200–0400 h when acupuncture was performed. Different color marks denote the respective MMPigs. Horizontal dotted lines denote the average of the respective data. *P < 0.05, **P < 0.01, between indicated pairs by the paired Student's *t*-test.

Sleep conditions of MMPigs were evaluated by the actigraph. As representative data for humans (48 years old, male) and MMPigs (MMPig 1), they were inactive for some hours in the nocturnal phase (Figure 3). The human and MMPig data showed 89.7 and 85.3% in SE; 323 and 307 min in TST; 7040 and 8996 in activity, respectively. As a result, MMPigs showed continuous high activity counts in the diurnal phase, indicating that MMPigs are diurnal animals. Sleep onset seems to correspond to lights off, since they were suddenly inactive at around 1900 h. MMPigs did not show any significant differences in activity counts for 20 min during treatment with the deep procedure at p-Baihui or Dafengmen, as compared with untreated MMPigs (p-Baihui, 6781 ± 286; Dafengmen, 6413 ± 756; untreated, 6850 ± 565). In clinical cases, acupuncture improves sleep conditions at night when the procedure is applied; therefore, we observed the effects of acupuncture on sleep conditions at night (2200–0400 h). As shown in Figure 3, slept at around 1900 and waking at around 0600 showed higher activity; therefore, we analyzed data during 2200–0400, corresponding to the middle of the lights-off period to evaluate sleep quality. As shown in Figure 4 as the results of experiment 1, MMPigs treated with a shallow or deep procedure at p-Baihui showed no significant differences in sleep conditions, including SE (46.1 ± 9.7; 43.7 ± 10.6%), TST (166.0 ± 35.0; 154.3 ± 37.9 min), and activity counts (29791 ± 6449; 28937 ± 7197), during 2200–0400 h from those of untreated MMPigs (SE, 48.7 ± 7.5%; TST, 175.4 ± 27.0 min; Activity counts, 24207 ± 4193). On the other hand, MMPigs treated with the deep procedure at Dafengmen showed significantly higher SE (63.6 ± 7.1%) and TST (229.0 ± 25.7 min) and lower activity counts (16895 ± 2872) during 2200–0400 h than those of untreated MMPigs; however, MMPigs treated with the shallow procedure at Dafengmen showed no significant differences in sleep-related parameters during 2200–0400 h, including SE (56.1 ± 6.0%), TST (202.1 ± 21.7 min), and activity counts (20543 ± 2770), compared with those of untreated MMPigs.

### 3.2. Urinary Catecholamine

In experiment 2, MMPigs treated with deep acupuncture at Dafengmen showed significantly higher TST during 2200–0400 h than untreated MMPigs (Table 1). MMPigs used in experiment 2 showed relatively lower SE, lower TST, and higher activity counts than MMPigs in experiment 1 before acupuncture treatment, probably due to the age difference. Under these conditions, MMPigs treated with the deep procedure at Dafengmen showed significantly lower values in norepinephrine in urine collected from 1900 to 0900 h than untreated MMPigs. On the other hand, MMPigs treated with the deep procedure at p-Baihui showed similar values in urinary norepinephrine to untreated MMPigs. There were no significant differences in urinary epinephrine among the three groups (Table 1).

## 4. Discussion

In the present study, we used the smallest pig available for animal experiments to evaluate the effects of acupuncture on sleep conditions using an actigraph and urinary catecholamine. Acupuncture has beneficial effects on sleep conditions only when treated with the deep procedure at an acupoint (Dafengmen) that is anatomically similar point to humans (Baihui, GV20). To the best of our knowledge, this is the first report describing the objectively beneficial effects of acupuncture on sleep conditions using large mammals.

Acupuncture has been widely applied clinically to people with sleep disturbances [7–10]; however, there are few objective data. One of the purposes of the present study was to establish an animal model to evaluate sleep conditions in order to obtain objective data on acupuncture procedures. For analysis of the effects of acupuncture on digestive function, rats and dogs have been used as an animal model [32–34]; however, rats are not diurnal animals and have different sleep conditions from humans. On the other hand, the MMPigs used in the present study are typical diurnal animals (Figure 3). Namely, MMPigs were inactive for a long period in the nocturnal phase and continuously active in the daytime. There is a long history of pigs coexisting with humans [18–20]; therefore, humans and pigs might have similar living patterns. Moreover, MMPigs, which have been developed recently in Japan, are small and placid, which are beneficial characteristics for an animal model. MMPigs adapted easily to the harness equipped with an actigraph, a device for evaluating sleep conditions. To obtain objective data on the effects of acupuncture, we used the actigraph for several successive days. As shown in Figure 4, untreated MMPigs showed 20–70% of SE, indicating that the living environment of MMPigs may be somewhat stressful. Namely, MMPigs are models of sleep disturbance. In addition, we measured urinary catecholamine during the nocturnal phase, which is an appropriate biomarker for sleep conditions [27–29]. Here, we used MMPigs, diurnal animals, equipped with an actigraph to evaluate the effects of acupuncture on sleep conditions.

The physiological mechanisms of acupuncture have been controversial, and hence the method of needling practices, optimal mode of stimulation, and selection of acupuncture points are complicated. In the clinical procedure for patients with sleep disturbances, Baihui is used as the acupuncture point [9, 10]. According to traditional Chinese medicine, Baihui is located at the vertex on the midline, so h-Baihui and p-Baihui are located at different points anatomically (Figure 1) [18–20]; therefore, we selected another acupoint, Dafengmen, which is anatomically similar to h-Baihui. As a result, only acupuncture at Dafengmen for a long duration improved SE, TST, and total activity during the night, and suppressed urinary excretion of norepinephrine. The duration of stimulation may be related to the beneficial effects. Moreover, these results mean that the acupoint for improvement of sleep quality is anatomically similar to h-Baihui (GV20) rather than p-Baihui determined by traditional Chinese medicine. In dogs, acupuncture at GV20 (located on the head) induced sedative effects based on EEG analysis, indicating that stimulation of GV20 by acupuncture may affect the central nervous system related to the hypothalamus [35–37]. On the other hand, stimulation of GV20 by acupuncture in rats increased locomotor activity [38], indicating that the effects of acupuncture at GV20 may be different outcomes in species. GV20 may be located in an area governed by the trigeminal nerve and/or greater occipital nerve in different species [35, 36]. Acupuncture stimulation may be transferred to hypothalamic parasympathetic nerve systems involved in sleep or wake conditions through trigeminal and/or greater occipital nerves because there is a crosstalk between nerve systems [39, 40]. Regarding the relationship between neurological anatomy and the physiological response against acupuncture, further experiments using MMPigs will be required.

In conclusion, we have established an animal model to evaluate the effects of acupuncture on sleep conditions. Beneficial effects occur when acupuncture is performed at a specific point, indicating that the effects may be induced by transferring the stimulus to the central nervous system, including the sleep center, through trigeminal and/or greater occipital nerves.

## Figures and Tables

**Table 1:** Effect of acupuncture at different acupoints on sleep conditions and urinary catecholamine.

	Untreated	p-Baihui	Dafengmen
SE (%)	31.8 ± 6.0	37.8 ± 8.5	51.9 ± 7.3
TST (min)	114 ± 22	136 ± 31	187 ± 27**
Activity (10^3^ × counts)	27.6 ± 1.3	27.7 ± 5.4	22.1 ± 3.7
Norepinephrine (ng)	870 ± 124	693 ± 352	467 ± 18*
Epinephrine (ng)	114 ± 36	168 ± 89	149 ± 36

Data are presented as the means ± SEM. SE, sleep efficiency; TST, total sleep time. *P < 0.05; **P < 0.01 versus untreated MMPigs.
